# Silver Diamine Fluoride as a Medicament for the Indirect Pulp Therapy in Primary Teeth: A Review of the Literature

**DOI:** 10.7759/cureus.60780

**Published:** 2024-05-21

**Authors:** Mohammed J Barry, Khlood Baghlaf, Najlaa Alamoudi

**Affiliations:** 1 Pediatric Dentistry, Department of Pediatric Dentistry, King Abdulaziz University, Jeddah, SAU

**Keywords:** sdf and hall technique, ipt material, dental liner, indirect pulp therapy, silver diamine fluoride

## Abstract

Silver diamine fluoride (SDF) has been demonstrated to be effective in arresting caries lesions and, recently, clinical trials have assessed the effectiveness of SDF as a medicament for indirect pulp therapy (IPT) in primary teeth. This review aims to summarize the literature related to the use of SDF and find out if SDF can be used as an effective material for IPT. A literature search was undertaken on electronic databases including PubMed, MEDLINE, ScienceDirect, and Google Scholar, which elicited 50 studies employing different materials in the IPT of primary molars; however, of them, only four clinical trials used SDF as indirect pulp capping (IPC) material. SDF has the potential to be a useful material for IPT in primary teeth. It is a handy choice for pediatric dentists due to its minimum invasiveness, ease of application, and ability to stop the progression of caries. However, more studies are needed to determine whether SDF can be used routinely for IPT and whether it can even replace the currently available materials, as well as to fully realize its potential and establish criteria for its ideal application in IPT procedures.

## Introduction and background

Indirect pulp therapy (IPT) is performed in teeth where the caries lesion is deep and close to the pulp chamber, without any evidence of pulp exposure [1]. It involves removing caries from the cavity walls and dentin-enamel junction while leaving caries in the cavity floor intact and covering it with a biocompatible material to create a biological seal [2]. To achieve optimal results in IPT, case selection is key. A proper diagnosis requires adequate clinical examination, radiographic evaluation, and dental history [3]. IPT has been proven to be more successful than conventional treatment in primary teeth [1,4,5]. Calcium hydroxide (CH) has been traditionally considered the finest material for IPT [6], thanks to its multiple advantages, such as cost-effectiveness, biocompatibility, and antimicrobial effect [7]. However, it is associated with a few disadvantages as well, as it may disintegrate over time, which will allow leakage [8]. Despite these drawbacks, CH is the material of choice for IPT. Other available materials for IPT include mineral trioxide aggregate (MTA), biodentine, and resin-modified glass ionomer cement (RM-GIC) [9].

Silver diamine fluoride (SDF), which was recently approved for use in the United States [10], has been demonstrated to be effective in preventing caries lesions [11]. It is a highly valuable therapy that can be incorporated into a patient's caries management plan. SDF-treated caries lesions typically turn black and hard [12]. This review aims to summarize the literature related to the use of SDF and find out if SDF can be used as an effective material for IPT. Additionally, this review summarizes the advantages and disadvantages of other available IPT materials, aiming to inform pediatric dentists about clinical facts related to the use of SDF as an indirect pulp capping (IPC) material in primary molars.

## Review

Methods

Data Sources and Search Strategy

A search was undertaken on electronic databases including PubMed, MEDLINE, ScienceDirect, and Google Scholar in December 2023. The search was designed to include studies in English only studies in this review. The following keywords were used to identify potentially relevant papers: (SDF) OR (silver diamine fluoride) OR (SMART technique) AND (Indirect pulp capping)) OR (indirect pulp treatment)) AND (primary teeth)) OR (children).

Results

The search elicited 255 articles from three different databases. After the removal of the duplicates, only 50 articles remained for screening. Finally, it was found that only four clinical trials used SDF in IPT. The review results showed that several materials are available for IPT.

Calcium Hydroxide (CH)

Calcium hydroxide (CH) has historically been considered the gold standard for IPT, with long-term observation of CH studies showing that it is not comparable to other bioactive materials [13]. CH is a biocompatible alkaline material that reduces bacterial infection and increases pulp dentine remineralization [14,15]. It still has several limitations, including poor sealing capability, disintegration over time, many tunnel defects in the generated dentin bridges, and insufficient adhesion to dentinal walls [16,17]. However, the main drawback of using CH as a pulp capping agent is its high solubility. The material often breaks down, failing to create a durable barrier against bacterial infection two years after application, and becomes weak in the reparative dentin under the capping material [8].

Mineral Trioxide Aggregate (MTA)

Mineral trioxide aggregate (MTA) was first developed in the early 1990s by Torabinejad et al. as a root-end filling and endodontic repair material [18]. It possesses good physical characteristics [19] and has demonstrated the ability to encourage mineralization where the pulp is exposed and may be able to preserve pulp vitality. Because of this, MTA's indications have significantly increased beyond its original use, and it has lately emerged as a superior alternative to CH in many more medical settings, such as direct pulp capping [20,21]. MTA can promote collagen production from cells when employed in IPT, which enables it to build dentine bridges of higher quality than calcium hydroxide [22]. The high cost and lengthy sitting times are the two main drawbacks of using the MTA [19,22].

Resin-Modified Glass Ionomer Cement (RM-GIC)

The development of resin-modified glass ionomer cement (RM-GIC) represents a significant technological development related to glass ionomers and has had a significant impact on pediatric dentistry [23]. Vitrebond (3M Corporation, St. Paul, MN) is an RM-GIC product [24, 25]. RM-GIC is well-known for decreasing postoperative sensitivity, as well as fluoride ion release, and antimicrobial effect [26-28]. Within the first 24 hours of insertion into a cavity preparation, RM-GICs have an initial pH of roughly 4.0-5.5. As a result, the glass ionomer demineralizes the nearby dentin, releasing ions and perhaps even the bioactive substances that were previously trapped. When there is still a layer of dentin between the substance and the pulp, the pulpal response to glass ionomer is favorable [9].

Biodentine

In 2011, the tricalcium silicate-based cement biodentine was introduced (Septodont, Saint-Maur-des-Fossé, France). This relatively new biomaterial, which is being investigated for use in vital pulp therapy procedures, claims to have qualities similar to MTA [8]. Biodentine was created as a permanent, biocompatible dentin replacement that could be used for final composite restoration in a single session [29]. Furthermore, it is a biocompatible material that promotes the formation of tertiary dentine via odontoblastic differentiation [30]. Also, it has effective antibacterial properties [31]. A recent randomized controlled trial (RCT) aiming to compare biodentine with CH on primary teeth with deep caries lesions as an IPT treatment of choice concluded that biodentine is an acceptable material to achieve good therapeutic results [32].

A summary of the above findings is presented in Table 1.

**Table 1:** A summary of materials used in indirect pulp capping of primary teeth

Material	Advantages	Disadvantages	Success rate
Calcium hydroxide	Biocompatible; antimicrobial activity; induction of calcified barrier; stimulates fibroblasts; inexpensive.	May dissolve after one year; poor sealing properties	94% [33]
Mineral trioxide aggregate	Biocompatible; antimicrobial activity; improved sealing properties; induced osteogenesis; promotes healing.	Discoloration; prolonged setting time; high cost	100% [34]
Resin-modified glass ionomer	Biocompatible; decreases postoperative sensitivity; fluoride ion release; antimicrobial effect	Initial pH is 4.0-5.5	96.5% [35]
Biodentine	Biocompatible; antimicrobial activity; increased marginal adaptation; high bond strength; formation of reparative dentin	High cost	98.3% [32]

Silver Diamine Fluoride (SDF)

Nishino and Yamaga pioneered the use of SDF in the 20th century. They conducted a laboratory study using SDF as a preventive technique for new caries and the findings showed that SDF reduced the severity of the lesion compared to the control group [36,37]. Later on, clinical studies on human participants to measure the preventive effects of SDF showed that it decreased the development of new caries lesions by 73% compared to the control group [38]. Multiple clinical trials have evaluated the effectiveness of SDF in the management of dental caries [12,36-38]. A number of those trials looked into the uses of SDF in caries arrest for primary molars and the incidence of developing new lesions, and they found significant results regarding the high effectiveness of SDF [39,40]. SDF is approved in the United Kingdom to treat dentin hypersensitivity and appears to be effective in treating sensitive MIH molars [41].

While studies on SDF and its ability to effectively seize caries progression have been conducted for decades, its exact mechanism remains an area of ongoing research [42]. There are different theories about the caries-inhibiting effect of SDF. One of these involves the obturation of dentinal tubules since caries is mostly spread by the tubules [43]. A different hypothesis has considered the results of the reaction between SDF and the mineral content of the tooth since dentin's resistance to decalcification was improved by fluoride, which also prevented acid from penetrating the dentin's deeper layers [44]. Additionally, there is an anti-enzymatic activity of the reaction products. The antibacterial property is brought on through dextran-induced agglutination and enzyme inhibition [44]. Matrix metalloproteinases (MMPs) are enzymes that break down extracellular components. Moreover, it has been suggested that MMPs, which are found in both saliva and dentin, are crucial in the emergence of dentin caries lesions [45].

According to the American Academy of Pediatric Dentistry (AAPD) chairside guide, the criteria for teeth selection are as follows: no report of spontaneous pain, no sign of pulpal inflammation, cavitated caries lesions close to the pulp, and any surface having cavitated caries lesions that can be brushed for SDF application [12]. The AAPD advises using 38% SDF as part of a comprehensive caries management strategy to prevent cavitated caries lesions in primary teeth [46].

Silver Diamine Fluoride and Dental Caries

The most frequently used material for arresting caries is SDF [47]. The effectiveness of SDF as a caries-preventing agent and an arresting agent has been the subject of several studies. Also, many studies have compared it to multiple other caries management methods. In 2002, a prospective controlled clinical trial involving children aged three to five years was conducted, aiming to compare the usage of four-yearly applications of either 38% SDF or 5% sodium fluoride (NaF). The researchers found that SDF has a 96% success rate in arresting caries compared to NAF's 41.3% [39]. A prospective controlled clinical trial in 2005 including children aged five to six years examined the effectiveness of applying 38% SDF solution twice a year to prevent caries in primary teeth; Its findings revealed that SDF had an efficacy rate of 97% for caries arrest after three years of follow-up [40].

In 2012, a randomized controlled trial compared the arresting effects of SDF and glass ionomer cement (GIC) on dental caries among children aged five to six years. At the six-month follow-up, they found that the arresting impact on children in the SDF group was 85% and it decreased to 66.9% at 12 months. This was greater than in the GIC group, which had 43% at six months and 38% at 12 months. After 12 months, the study also showed that SDF was 1.73 times more effective in arresting caries than GIC [48]. A randomized clinical trial on the arresting effects of three different topical fluoride application techniques was conducted in 2016 (annual application of SDF, three-time application of SDF, or three-time application of 5% NaF varnish) on primary teeth of children aged three to four years. The study's findings indicate that at 18 months, caries arrest rates were higher in the SDF (40-35%) groups compared to the 5% NaF varnish (27%) group [49].

Side Effects of Silver Diamine Fluoride

In terms of biocompatibility, silver is very compatible with the human body, and it is used in a variety of medical devices [50-53]. Nevertheless, its excess use can be harmful to the body, as it can accumulate in the skin, liver, kidneys, gingival, and nails [54,55]. However, there is a lack of significant data about the probable toxic effects of silver [56]. Several reports on the use of silver in dental clinics and SDF indicate that when they come into contact with the gingiva, patients may experience a mild increase in erythema, and small white lesions in the mucosa may appear [40, 57]. However, the material's caries-arresting ability may outweigh this minor discomfort.

The most noticeable side effect of SDF is the black discoloration of caries on the teeth [58,59]. Pulpal irritation is also considered one of the side effects of SDF along with oral soft tissue irritation and dental staining [60]. An in vitro study reported that adding potassium iodide to SDF before its application could lessen tooth discoloration [61]. According to a systematic review by Contreras et al. (2017), using lower concentrations of SDF might help with reducing the onset of SDF side effects, albeit at the expense of reducing its effectiveness in arresting caries [62].

Silver Diamine Fluoride as an Indirect Pulp Therapy Material in Primary Teeth

The use of SDF in various dental applications is gaining more popularity, and it has shown significant results as a noninvasive treatment method for arresting and preventing caries [63]. SDF has various properties, including raising the biofilm's pH, antimicrobial action, and reducing dentin demineralization [64]. These along with the ease of use, and the fact that it can be applied in uncooperative patients and patients with special healthcare needs (SHCN) [62], make it a very attractive option among dentists. A recent systematic review by Baghlaf et al. (2023) concluded that SDF showed comparable clinical and radiographic outcomes as CH as an indirect pulp material [65]. In 2020, an in vivo study aimed to compare SDF and CH as IPT materials; it found that the rate of clinical and radiographic success of using 38% SDF was 100% after one-month follow-up, with no noticeable difference between the two groups [66].

Table 2 shows a comparison of clinical trials using SDF in indirect pulp capping in primary teeth. 

**Table 2:** Comparison of clinical trials that used SDF in indirect pulp capping in primary teeth MTA: mineral trioxide aggregate; RM-GIC: resin-modified glass ionomer cement; SDF: silver diamine fluoride

Study	Control	Sample size	SDF technique	Follow-up period	Success rate of SDF as IPT (success of control)
Shah et al., 2020 [66]	Calcium hydroxide	34 primary anterior and posterior teeth, randomly classified into two groups	Caries excavation - cavity was dried with cotton pellets - 38% SDF was applied at the base of the cavity for 3 minutes - rinsing for 30 seconds. After drying, the cavity was restored with GIC Fuji IX	1 week, 1 month	100% (93.75%)
Divyashree, 2021 [67]	1: MTA, 2: Dycal	25 patients in each group	Caries removal, SDF was applied with a micro tip applicator for 15 sec to the floor of the cavity. Following the placement of the test materials the tooth was subsequently restored with RM-GIC	1, 3, 6 months	Not reported
Patil et al., 2021 [68]	Calcium hydroxide	50 primary molars, 25 in each group	The applicator tip was wetted with SDF solution and applied over the deepest layer of the cavity for 2 minutes - removed excess with a cotton palate - dried and RMGI was applied as the final restoration	3, 6 months	96% (92%)
Shafi et al., 2022 [69]	Calcium hydroxide	20 patients in each group	Washing and drying the cavity - two or more drops of diluted SDF for 2 minutes - washing and drying the cavity	12 months	96% (91.6%)

According to a study conducted to assess the success rate of SDF, MTA, and Dycal as IPT material on primary teeth, where they placed the materials in teeth and restored with RM-GIC, the results after a six-month follow-up period showed that SDF had arrested further caries progression, provided adequate biological seal, without any adverse reactions to the pulp. However, after six months, the Dycal group had the most reparative dentin with an average of 0.15 mm, followed by the MTA group with 0.11, while the SDF group had 0.07 [67].

An RCT compared the use of SDF and calcium hydroxide as IPT on primary teeth, with 50 primary molars, 25 for each group, and a follow-up period of three and six months. The results showed a 96% success rate for the SDF group and an 88% success rate for the calcium hydroxide group in the same time frame. However, there was no statistically significant difference between the two groups [68]. Shafi et al. (2022) conducted an RCT to evaluate diluted SDF against calcium hydroxide with a light cure used as an IPC material in primary molars. With a sam of 20 patients in each group and a 12-month follow-up period, the results supported the use of diluted SDF with a 96% success rate, whereas the light-cured calcium hydroxide had a success rate of 91.67% [69].

Silver Diamine Fluoride With Modified Hall Technique

The Hall technique is one of the newest techniques for preventing caries in primary molars [70]. It can arrest dental caries and protect primary teeth using simple biological principles; by using this technique, the caries lesion with the super facial plaque layer is sealed, and biofilm will be changed to a less cariogenic flora, slowing down caries' progress [71]. In addition, with its several advantages such as its noninvasive nature [72], being a quick procedure [73], cost-effectiveness [74], and the fact that it can be performed in a single visit [75], it has become a widely used method in treating primary molars in children. A 2022 study aimed to look at increasing the success rate of the Hall technique by eliminating bacteria present in caries in the lesion by using SDF before crown placement; the results showed no statistically significant findings; however, the success rate when adding SDF was 96.2% while it was 88.7% when only the Hall technique was used [76].

Light-Cured Silver Diamine Fluoride

An ex vivo study found that using light cure after SDF application added in the precipitation of more silver ions into infected dentin [77], A recent systematic review compared the use of light cure with SDF to SDF without light cure. The researchers concluded that light curing with SDF can be a beneficial method for increasing SDF's clinical success [78]. Although the use of dental light cure with SDF is considered a relatively new technique, it has shown promising results; however, more clinical trials need to be conducted to confirm its efficacy.

Discussion 

In pediatric dentistry, IPC is an essential procedure for treating severe caries lesions close to the pulp chamber without exposing the pulp. The medication selected for IPT has a significant impact on how well patients respond to treatment. CH has long been the gold standard for IPT because of its antibacterial and biocompatibility qualities [14,15]. However, more recently, materials with better sealing qualities and biocompatibility have been introduced, such as MTA, resin-modified glass ionomer, and biodentine. SDF is a versatile substance that has attracted great interest in dentistry, especially due to its effective caries lesion prevention and arrest capabilities. Studies indicate that SDF holds promise as an IPT material for primary teeth, with benefits including low patient discomfort, antibacterial activity, and simplicity of use [39,48]. SDF is a useful addition to a pediatric dentist's armament because it has also demonstrated a high success rate in inhibiting the advancement of dental cavities.

Studies that have compared SDF to more conventional IPT materials like MTA and calcium oxide have highlighted the effectiveness of SDF. Comparable, if not higher, clinical and radiographic success rates have been demonstrated by SDF in preventing caries and promoting pulp repair. Furthermore, advancements like light-cured SDF and the modified Hall technique with SDF have demonstrated encouraging outcomes in terms of improving the efficacy of SDF in IPT operations [66-69].

Despite its demonstrated efficacy and various beneficial aspects, SDF is also associated with several drawbacks and side effects, such as tooth discoloration and possible pulpal irritation [58,59]. Nevertheless, these disadvantages are surpassed by the material's significant benefits in the control of dental caries, particularly in younger patients in whom non-invasive methods are generally favored [40,48].

To sum up, SDF has the potential to be a highly useful material for IPT in primary teeth. It is a useful choice for pediatric dentists due to its minimally invasive nature, ease of application, and ability to stop the progression of caries. Further research is required to fully realize its potential and establish criteria for its ideal application in IPT procedures. Future studies should focus on determining if SDF can be used routinely for IPT and whether it can even replace the currently available materials.

## Conclusions

SDF offers excellent advantages in the field of noninvasive pediatric dentistry through its ability to successfully arrest the progression of dental caries. According to various studies, SDF shows promising results and outcomes as an IPT material in primary teeth. However, more research is needed to establish if SDF can be used routinely for IPT and if it can even replace the currently available materials. Our findings highlight the need for further clinical trials with larger sample sizes, focusing on the use of light cure after SDF application.
